# Green tea polyphenols, epicatechin gallate and epigallocatechin gallate, alleviated aberrant metabolic changes caused by olanzapine in rats

**DOI:** 10.22038/ajp.2025.25926

**Published:** 2025

**Authors:** Elmira Hoseini, Bibi Marjan Razavi, Mohaddeseh Sadat Alavi, Hossein Hosseinzadeh, Ali Roohbakhsh

**Affiliations:** 1 *Department of Pharmacodynamics and Toxicology, School of Pharmacy, Mashhad University of Medical Sciences, Mashhad, Iran*; 2 *Pharmaceutical Research Center, Pharmaceutical Technology Institute, Mashhad University of Medical Sciences, Mashhad, Iran*; 3 *Pharmacological Research Center of Medicinal Plants, Mashhad University of Medical Sciences, Mashhad, Iran*; 4 *Department of Pharmacology, Faculty of Medicine, Mashhad University of Medical Sciences, Mashhad, Iran*

**Keywords:** Olanzapine, Metabolic syndrome, Green tea, Epigallocatechin gallate, Epicatechin gallate

## Abstract

**Objective:**

Olanzapine, a well-known antipsychotic drug, causes considerable weight gain and metabolic abnormalities in patients. Green tea (*Camellia sinensis*) has anti-obesity, antihypertensive, antihyperlipidemic, and anti-diabetes effects. The aim of this study was to investigate the potential effects of epicatechin gallate (ECG) and epigallocatechin gallate (EGCG) as green tea polyphenols on metabolic changes induced by olanzapine.

**Materials and Methods:**

We used fourteen groups of rats and subjected them to intraperitoneal injections once a day for eleven days: 1: Control. 2: Olanzapine (5 mg/kg/day). 3, 4, and 5: Olanzapine + EGCG (10, 20, and 40 mg/kg/day, respectively). 6, 7, and 8: EGCG (10, 20, and 40 mg/kg/day, respectively). 9, 10, and 11: Olanzapine + ECG (10, 20, and 40 mg/kg/day, respectively). 12, 13, and 14: ECG (10, 20, and 40 mg/kg/day). The body weights were recorded every three days and food consumption was evaluated every day. At the end of the study, lipid profile, systolic blood pressure (SBP), leptin and fasting blood sugar (FBS) levels, and locomotor activity were assessed.

**Results:**

Olanzapine considerably increased weight, food intake, triglycerides, low-density lipoprotein (LDL), cholesterol, SBP, leptin, and FBS, and decreased high-density lipoprotein (HDL), and locomotor activity. Co-administration of ECG or EGCG at different doses significantly suppressed olanzapine-induced weight gain, and elevated plasma lipids, SBP, leptin, and FBS levels. Both compounds also considerably increased locomotor activity and HDL levels.

**Conclusion:**

These findings suggest that ECG and EGCG could be promising adjunct therapies to counteract the metabolic side effects of olanzapine.

## Introduction

A new generation of antipsychotics, known as atypical antipsychotics, has been used as first-line medications prescribed to treat a broad range of mental health issues involving schizophrenia and mania-depressive disorders (Xu and Zhuang, 2019). These medications effectively control positive, negative, and cognitive symptoms in schizophrenia patients and have a lower potential to induce extrapyramidal symptoms than classic antipsychotics (Urban and Cubała, 2017). These drugs, however, can lead to metabolic syndrome including obesity, hyperlipidemia, hyperglycemia, and elevated blood pressure (Carli et al., 2021). According to a study, almost one in three schizophrenia patients meet metabolic syndrome criteria. Metabolic syndrome occurs in about 40% of chronic schizophrenic patients and is related to reduced life expectancy and poor patient compliance (Carli et al., 2021). The rate of morbidity and mortality is higher in schizophrenic individuals than in healthy people, and metabolic syndrome becomes a major concern (Nguyen et al., 2018). 

Olanzapine is an atypical antipsychotic medication belonging to the dibenzothiazepine class that blocks serotonin receptors with little effect on dopamine transmission. Similar to other atypical antipsychotics, olanzapine use has been connected with various metabolic effects such as weight gain, accumulation of visceral fat, hyperlipidemia, increased blood leptin levels, insulin resistance, diabetes type 2, and high blood pressure (Akinola et al., 2023; Li et al., 2019; Wu et al., 2022). About 50% of patients treated with olanzapine for more than one month are obese, showing an extremely high prevalence (Lieberman et al., 2005). The goal is to find new strategies to prevent and/or reduce the metabolic abnormalities induced by olanzapine (Al-Naimi et al., 2019; Hu et al., 2014). A combination of olanzapine and an opioid receptor antagonist, samidorphan, has recently been authorized for treating schizophrenia and mania-depression. In comparison to olanzapine, this combination could lead to weight loss and reduced waist circumference, and it was tolerated well (Correll et al., 2020). However, some researchers believe that the weight change induced by olanzapine/samidorphan is not much different from olanzapine alone (Monahan et al., 2022). 

 Herbal medications help manage metabolic syndrome caused by olanzapine (Al-Naimi et al., 2019; Ardakanian et al., 2022). Many health benefits are associated with green tea (*Camellia sinensis*) and its polyphenolic components, including antioxidant, antihyperglycemia, antihyperlipidemia, and anticancer properties. Green tea consumption is considered beneficial for reducing cardiovascular disease risk in traditional medicine (Bedrood et al., 2018; Chacko et al., 2010). Epigallocatechin gallate (EGCG) found in green tea leaves has beneficial effects on glucose and lipid metabolism. Moreover, it augments the metabolic and vascular effects of insulin and increases fatty acid metabolism in skeletal muscle (Chen et al., 2011; Esmaeelpanah et al., 2021). EGCG enhances endothelial cell function, improves glucose tolerance and insulin sensitivity, and decreases blood pressure. It also has protective effects against myocardial injury in spontaneously hypertensive rats (Potenza et al., 2007). Epicatechin gallate (ECG), another polyphenol of green tea, has anti-atherosclerotic properties (Yu et al., 2021). It inhibits hyperlipidemia and oxidative stress and protects against cardiac ischemia/reperfusion damage (Qi et al., 2019; Rameshrad et al., 2017). 

The present study aimed to identify the protective effect of ECG and EGCG on experimental metabolic syndrome induced by olanzapine in male rats by evaluating weight gain and food intake. Additionally, fasting blood sugar (FBS), plasma lipids, and leptin levels, blood pressure, and locomotor activity were evaluated.

## Materials and Methods

### Animals

Male Wistar rats (180 g, 7 weeks old) were obtained from the animal center of the School of Pharmacy (Mashhad University of Medical Sciences). In each cage, there were five animals. The animals were kept on a 12-hr light/dark cycle without restrictions on food and water. For measuring factors such as FBS, lipids, and leptin, animals had to fast for 12 hr before taking a blood sample. 

### Materials

Olanzapine powder was obtained from Sobhan Darou (Iran). ECG and EGCG Green tea catechins were obtained from Anhui Minmetals Development Import & Export (China). Olanzapine solution was prepared using sterile normal saline 0.9% (NS) and two drops of acetic acid. ECG and EGCG were dissolved in sterile NS and prepared freshly each day. The Rat Leptin ELISA Kit was purchased from Abcam (ab100773, UK). Low-density lipoprotein (LDL), high-density lipoprotein (HDL), triglyceride (TG), total cholesterol, and FBS kits were purchased from Pars Azmun (Iran).

### Groups

After acclimatization, the rats were randomly separated into 14 groups (n=5). Animals were treated intraperitoneally once a day for eleven days. 

1: Animals that were given olanzapine solvent (control), 

2: Animals that received olanzapine (5 mg/kg/day), 

3, 4, and 5: Animals that received EGCG (10, 20, and 40 mg/kg/day, respectively) concomitant with olanzapine (5 mg/kg/day), 

6, 7, and 8: Animals that received EGCG (10, 20, and 40 mg/kg/day, respectively), 

9, 10, 11: Animals that received ECG (10, 20, and 40 mg/kg/day, respectively) concomitant with olanzapine (5 mg/kg/day), 

12, 13, and 14: Animals that received ECG (10, 20, and 40 mg/kg/day, respectively).

### Food intake and weight gain measurement

During the study, body weights were calculated every three days, and food intake was measured daily (Ardakanian et al., 2022). 

### Plasma metabolic parameters measurement

After 12 hr of fasting, rats were sacrificed on the last day. The serum levels of FBS, plasma lipids including TG, total cholesterol, HDL, LDL, and leptin in the serum were measured spectrophotometrically by ELISA kits (Hitachi, Japan) (Malekzadeh et al., 2019).

### Measuring systolic blood pressure (SBP)

A non-invasive tail-cuff method was used to measure rats' systolic blood pressure at the end of the study (Patil et al., 2006). A non-invasive blood pressure )NIBP( device (AD Instrument, Australia) was used for blood pressure measurement. After five measurements of SBP, mean SBP was determined.

### Locomotor activity measurement

On the last day, locomotor movements were calculated using an open-field test (Etemad et al., 2020). The open-field apparatus was a Plexiglas box (90 cm× 90 cm× 30 cm). The surface was divided into 25 equal-sized unit squares (18 cm × 18 cm). The rats were gently placed in the center of the device and allowed to explore freely for five minutes. Movement was calculated as the number of crossings between squares (Souri et al., 2024). Using 10% ethanol, the apparatus floor was cleaned and dried after each test.

### Statistical analysis

Data is reported as mean ± SEM. To compare the groups, a one-way analysis of variance (ANOVA) was used. Tukey was used for *post-hoc *analysis. A significant difference was defined as a p-value of less than 0.05.

## Results

### ECG and EGCG reversed mean food intake and weight gain increased by olanzapine

**Figure 1:**
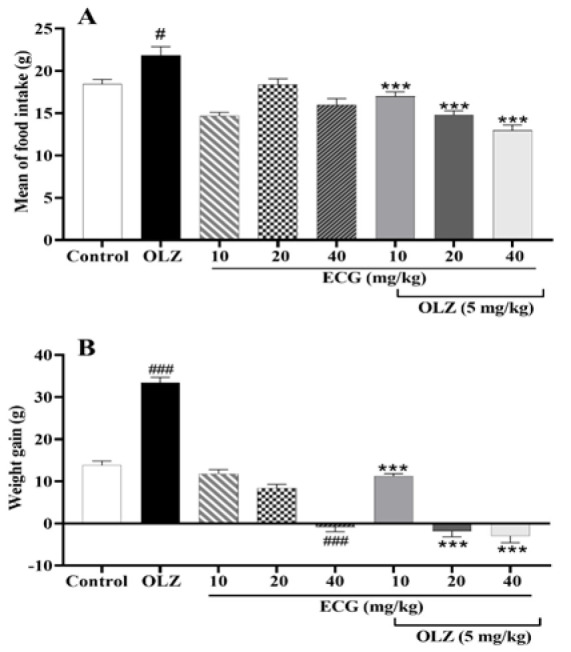
The effect of olanzapine 5 mg/kg, and ECG at 10, 20, and 40 mg/kg alone or in combination with olanzapine on A) mean food intake and B) weight gain in rats. Data is reported as mean ± SEM (n=5). #p<0.05 vs. control group, ***p<0.001 vs. olanzapine group. OLZ: olanzapine

As shown in Figure 1A, olanzapine (5 mg/kg) enhanced food consumption (p<0.05). Concomitant administration of olanzapine (5 mg/kg) and ECG (10, 20, and 40 mg/kg) decreased food intake (p<0.001). Figure 1B shows no statistically significant differences in body weight among different groups at baseline (p>0.05). Administration of olanzapine (5 mg/kg) significantly enhanced weight gain (p<0.001). Treatment with 10 and 20 mg/kg of ECG had no significant effect on weight gain, while ECG 40 mg/kg induced weight loss (p<0.001). Moreover, co-administration of all ECG doses with olanzapine, especially 20 and 40 mg/kg, caused remarkable weight loss compared to the olanzapine group (p<0.001). 

**Figure 2:**
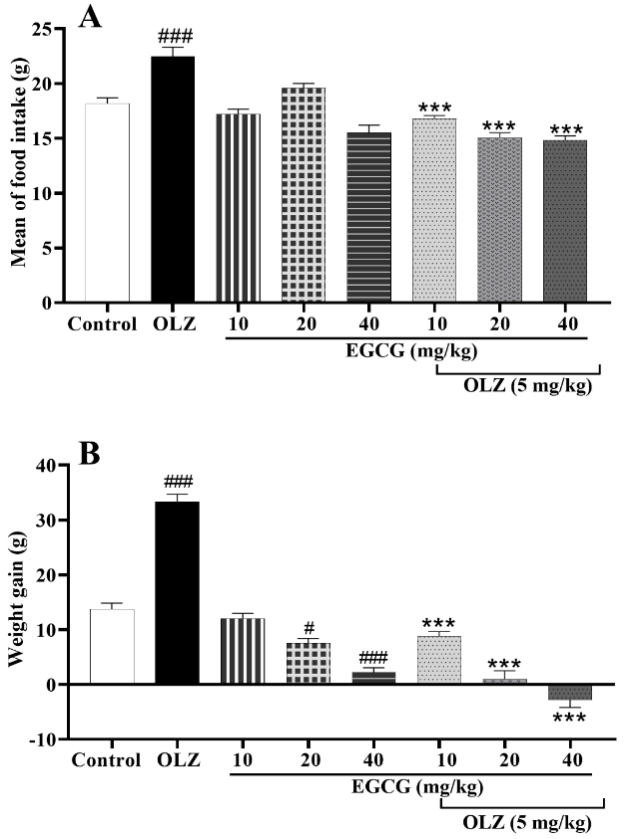
The effect of olanzapine 5 mg/kg, and EGCG at 10, 20, and 40 mg/kg alone or in combination with olanzapine on A) mean food intake and B) weight gain in rats. Data is reported as mean ± SEM (n=5). #p<0.05 and ###p<0.001 vs. control group, ***p<0.001 vs. olanzapine group. OLZ: olanzapine, EGCG: epigallocatechin gallate.

Furthermore, when EGCG (10, 20, and 40 mg/kg) was co-administered with olanzapine, mean total food intake decreased at all doses (p<0.001, Figure 2A). Administration of EGCG alone induced no significant change in food consumption. However, EGCG at 20 and 40 mg/kg reduced weight gain (p<0.001, Figure 2B). Co-administration of EGCG (10, 20, and 40 mg/kg) with olanzapine promoted significant weight loss compared to olanzapine-treated animals (p<0.001, Figure 2B).

### ECG and EGCG improved the aberrant lipid profile induced by olanzapine

**Figure 3:**
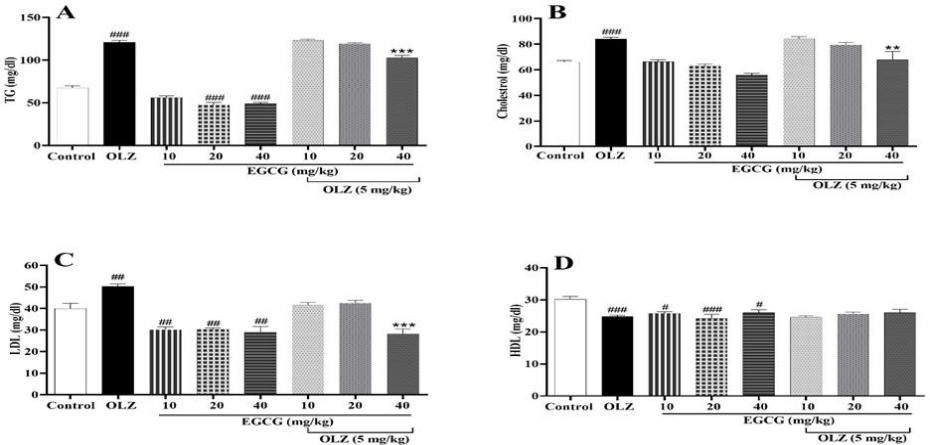
The effect of olanzapine 5 mg/kg, and ECG at 10, 20, and 40 mg/kg alone or in combination with olanzapine on A) TG, B) cholesterol, C) LDL, and D) HDL in rats. Data is reported as mean ± SEM (n=5). #p<0.05, ##p<0.01, ###p<0.001 vs. control group, *p<0.05, **p<0.01, and ***p<0.001 vs. olanzapine group. OLZ: olanzapine, ECG: epicatechin gallate

**Figure 4:**
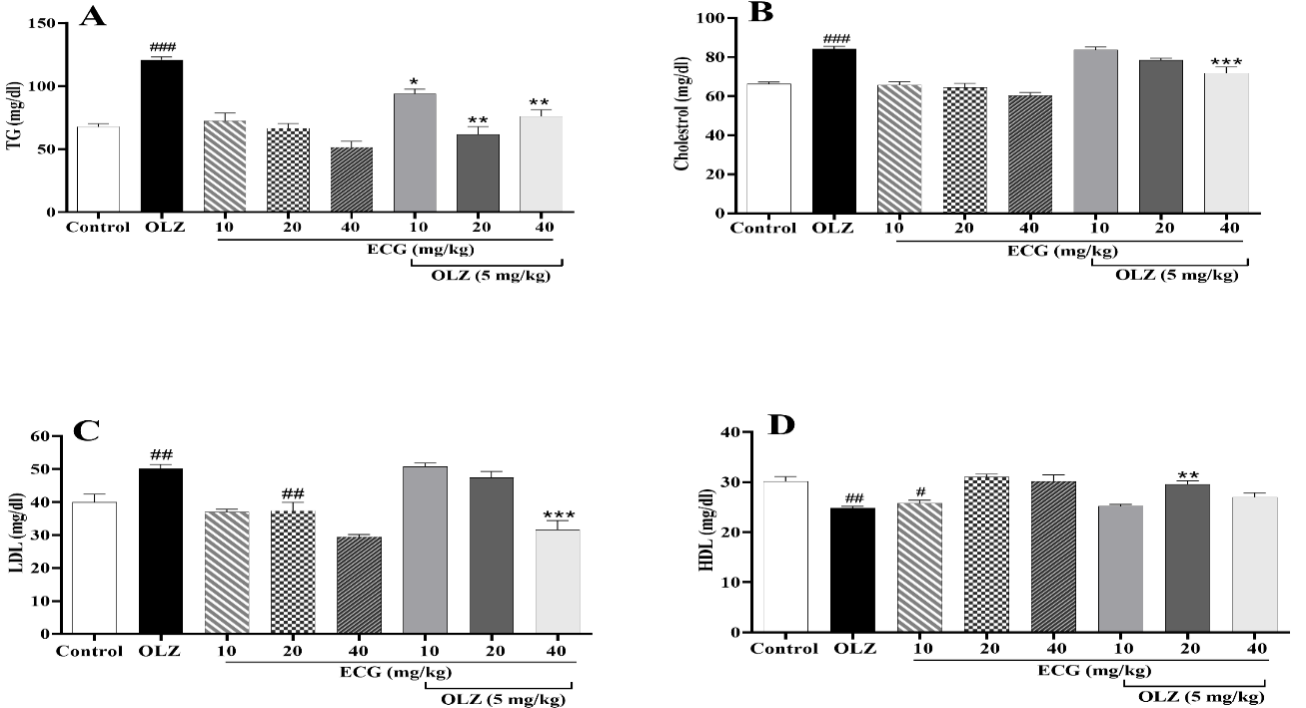
The effect of olanzapine 5 mg/kg, and EGCG at 10, 20, and 40 mg/kg alone or in combination with olanzapine on A) TG, B) cholesterol, C) LDL, and D) HDL in rats. Data is reported as mean ± SEM (n=5). #p<0.05, ##p<0.01, ###p<0.001 vs. control group, *p<0.05, **p<0.01, and ***p<0.001 vs. olanzapine group. OLZ: olanzapine, EGCG: epigallocatechin gallate.

The results revealed that in the olanzapine-treated group, TG, cholesterol (p<0.001, Figures 3A and 3B), and LDL (p<0.01, Figure 3C) levels were noticeably higher than the control group. Besides, olanzapine reduced HDL levels compared to control animals (p<0.001, Figure 3D). As illustrated in Figure 3, treatment with ECG in combination with olanzapine substantially reduced serum TG levels at all doses (p*<*0.01, Figure 3A). Concomitant administration of olanzapine and 40 mg/kg of ECG reduced LDL and cholesterol levels significantly (p*<*0.001, Figures 3C and 3D) compared to the olanzapine group. Also, ECG enhanced HDL levels at 20 mg/kg (p*<*0.001, Figure 3D). Similarly, EGCG at the dose of 40 mg/kg reduced serum TG, cholesterol, and LDL levels compared to the olanzapine group (p*<*0.001, Figures 4A, 4B, and 4C). However, it did not change HDL levels mitigated by olanzapine (Figure 4D).

### ECG and EGCG reduced SBP raised by olanzapine

**Figure 5:**
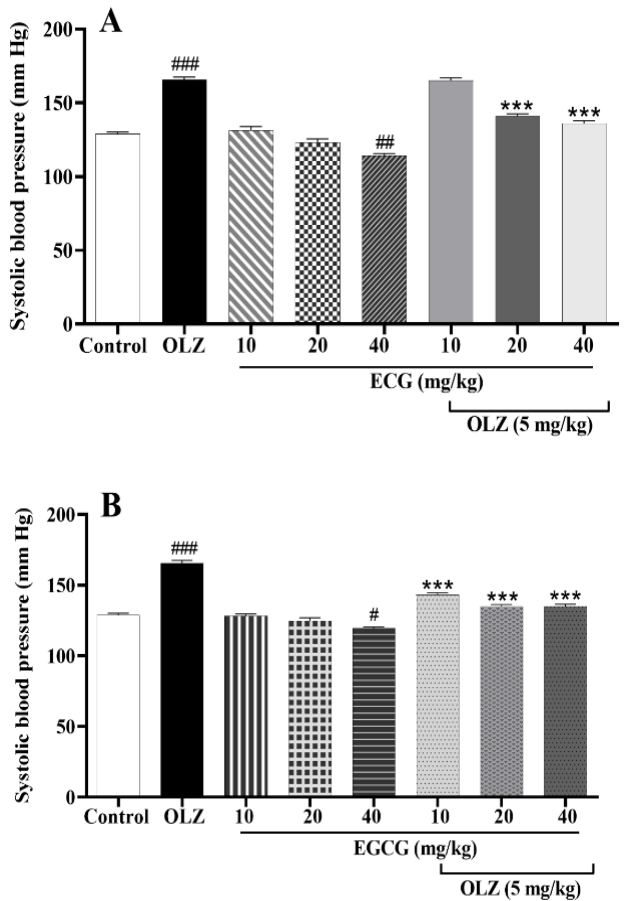
The effect of olanzapine 5 mg/kg, A) ECG 10, 20, and 40 mg/kg alone or in combination with olanzapine, B) EGCG at 10, 20, and 40 mg/kg alone or in combination with olanzapine on mean systolic blood pressure in rats. Data is reported as mean ± SEM (n=5). #p<0.05, ##p<0.01, ###p<0.001 vs. control group, ***p<0.001 vs. olanzapine group. OLZ: olanzapine, ECG: epicatechin gallate, EGCG: epigallocatechin gallate.

Mean SBP was significantly higher in rats receiving 5 mg of olanzapine than in the control group (p*<*0.001). A dose of 40 mg/kg of ECG decreased SBP. There was a significant decrease in mean SBP in rats treated with ECG (20 and 40 mg/kg) and olanzapine (p*<*0.001) in comparison to the olanzapine-treated group (Figure 5A). 

As shown in Figure 5B, all doses of EGCG reduced SBP increased by olanzapine (p*<*0.001). Moreover, administering 10 or 20 mg/kg of EGCG did not change the mean SBP. However, 40 mg/kg of EGCG reduced mean SBP compared to control animals (p*<*0.05, respectively).

### ECG and EGCG attenuated olanzapine-induced leptin enhancement

**Figure 6:**
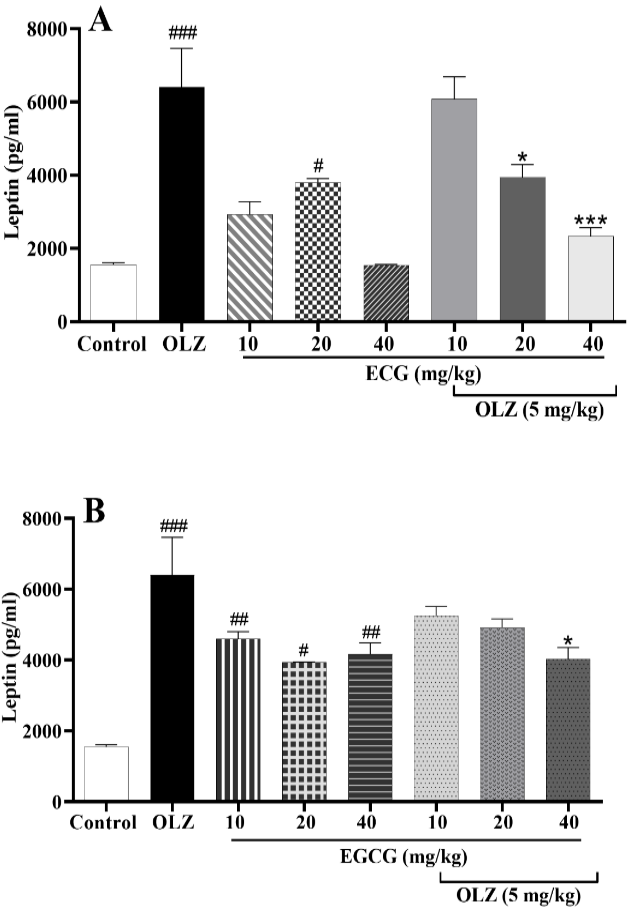
The effect of olanzapine 5 mg/kg, A) ECG at 10, 20, and 40 mg/kg alone or in combination with olanzapine, B) EGCG at 10, 20, and 40 mg/kg alone or in combination with olanzapine on leptin serum levels in rats. Data is reported as mean ± SEM (n=5). #p<0.05, ##p<0.01, ###p<0.001 vs. control group, *p<0.05 and ***p<0.001 vs. olanzapine group. OLZ: olanzapine, ECG: epicatechin gallate, EGCG: epigallocatechin gallate.

Leptin levels in the plasma were higher in the olanzapine group than the control animals (p*<*0.001, Figure 6). Leptin levels were significantly reduced after treatment with ECG (p*<*0.01, Figure 6A). Moreover, leptin levels were mitigated in animals receiving 40 mg/kg of EGCG (p*<*0.05, Figure 6B). Both ECG and EGCG reduced leptin levels when administered alone (p<0.05 and p<0.01, respectively, Figure 6).

### ECG and EGCG prevented glucose level enhancement induced by olanzapine

**Figure 7:**
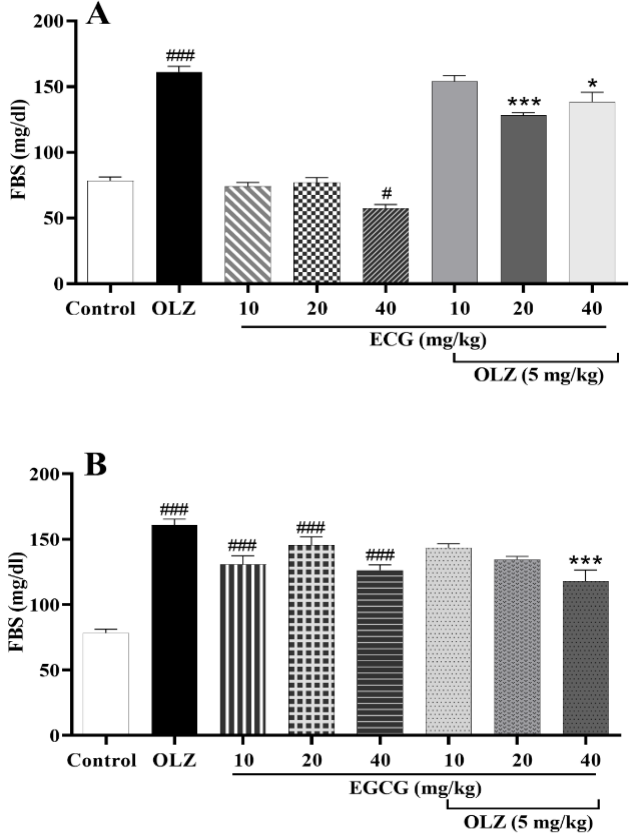
The effect of olanzapine 5 mg/kg, A) ECG 10, 20, and 40 mg/kg alone or in combination with olanzapine, B) EGCG at 10, 20, and 40 mg/kg alone or in combination with olanzapine on fasting blood sugar (FBS) level in rats. Data is reported as mean ±SEM (n=5). #p<0.05, ###p<0.001 vs. control group, *p<0.05 and ***p<0.001 vs. olanzapine group. OLZ: olanzapine, ECG: epicatechin gallate, EGCG: epigallocatechin gallate.

As compared to the control animals, the FBS level of olanzapine-treated animals was significantly higher (p*<*0.001, Figure 7). Although 40 mg/kg of ECG treatment reduced FBS (p*<*0.05, Figure 7A), the EGCG-treated group exhibited higher FBS levels than the control rats (p*<*0.001, Figure 7B). Animals that received either 20 or 40 mg of ECG plus olanzapine had lower FBS levels than the olanzapine group (p*<*0.001, Figure 7A). The results also showed a remarkable decrease in the FBS of animals treated with 40 mg of EGCG + olanzapine (p*<*0.001, Figure 7B) compared to the olanzapine-treated group. 

### ECG and EGCG improved locomotor activity impaired by olanzapine

**Figure 8:**
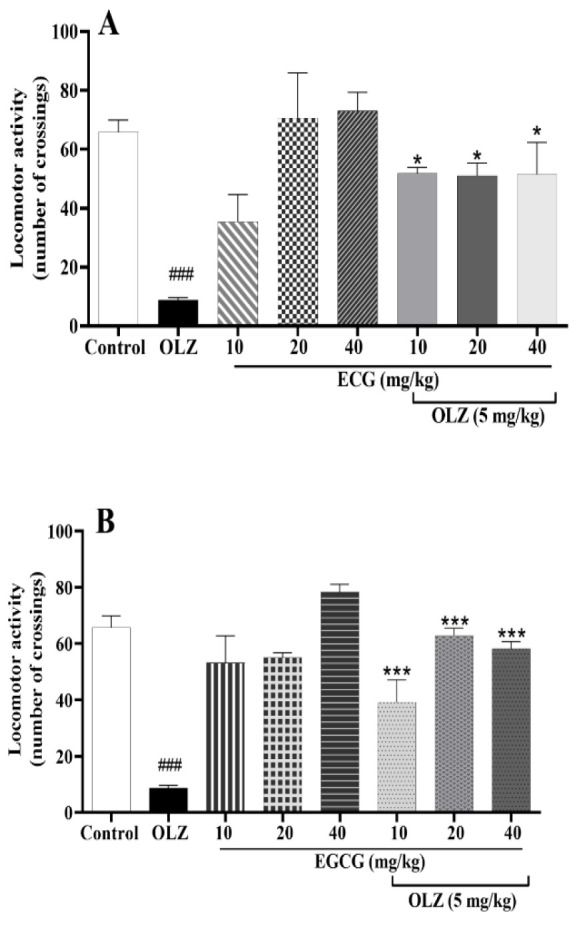
The effect of olanzapine 5 mg/kg, A) ECG 10, 20, and 40 mg/kg alone or in combination with olanzapine, B) EGCG at 10, 20, and 40 mg/kg alone or in combination with olanzapine on locomotor activity in rats. Data is reported as mean ±SEM (n=5). ###p<0.001 vs. control group, *p<0.05 and ***p<0.001 vs. olanzapine group. OLZ: olanzapine, ECG: epicatechin gallate, EGCG: epigallocatechin gallate.

As shown in Figure 8A, olanzapine treatment reduced movements (p<0.001). Locomotion was restored considerably in animals treated with ECG (10, 20, and 40 mg/kg) and olanzapine compared to the olanzapine group (p*<*0.05). Moreover, all doses of EGCG (10, 20, and 40 mg/kg) significantly increased movement compared to the olanzapine group as seen 

in Figure 8B (p*<*0.001). Treatment with ECG or EGCG did not affect locomotor activity at all doses tested in this study. 

## Discussion

In the present research, the protective potential of ECG and EGCG, two important polyphenols of green tea, against olanzapine-caused metabolic syndrome was determined in an experimental model. Our data revealed that ECG and EGCG prevented weight gain, dyslipidemia, elevated SBP, increased leptin levels, high blood sugar, and motor impairment induced by olanzapine.

Administration of olanzapine to rats enhanced food consumption and weight gain substantially, which is consistent with previous studies (Ardakanian et al., 2022; Malekzadeh et al., 2019; Razavi et al., 2020; Razavi et al., 2017). Metabolic syndrome and weight gain are two key reasons for discontinuing atypical antipsychotics, in particular, olanzapine (Meyer et al., 2005; Parabiaghi et al., 2016). Olanzapine increases blood pressure, and the levels of TG, LDL, blood glucose, and leptin. It also decreases HDL levels (Razavi et al., 2017). In accordance, it was indicated that chronic olanzapine administration promoted significant weight gain and fat accumulation (Coccurello et al., 2006). Weight gain caused by olanzapine has been linked to its antihistaminergic properties and subsequent hyperphagia or enhanced eating (Weston-Green et al., 2011). ECG and EGCG administration reduced food intake and decreased weight gain in olanzapine-treated rats. In agreement, spontaneously hypertensive rats treated with EGCG for 21 days had a moderate reduction in food intake and weight compared to enalapril-treated rats (Potenza et al., 2007). Likewise, EGCG treatment notably diminished overweighting in C57BL/6J mice supplemented with a high-fat/Western-style regime (Chen et al., 2011). In addition, green tea promoted weight loss in a mouse model of metabolic syndrome caused by a high-fat intake (Mei et al., 2023). Numerous preclinical and clinical studies have confirmed that olanzapine induces dyslipidemia (Li et al., 2020; Liu et al., 2015). Olanzapine increases fatty acids, TG, cholesterol, and LDL contents and decreases HDL levels (Ersland et al., 2019; Garman et al., 2007; Zugno et al., 2012). Daily administration of 3 mg/kg olanzapine for four weeks enhanced the whitening of the brown adipose tissue in mice. Furthermore, olanzapine exacerbates atherosclerosis by interfering with hepatic lipid metabolism, triggering hyperlipidemia, and promoting aortic inflammation. In apoE-knockout mice treated with olanzapine, liver lipid accumulation was increased remarkably (Chen et al., 2018). In that study, dyslipidemia elicited by olanzapine was improved by concomitant ECG and EGCG administration. Similar to our findings, EGCG treatment significantly improved the lipid profile. In addition, it decreased plasma alanine aminotransferase level, fatty liver incidence, and liver injury, and suppressed inflammatory cytokines in mice supplemented with a high-fat/Western-style diet (Chen et al., 2011). In a separate study, we found that 25-100 mg/kg/day of green tea extract protected rats against metabolic syndrome caused by olanzapine (Razavi et al., 2017). There have been reports of the same findings elsewhere (Lukitasari et al., 2020; Saifur Rohman et al., 2021). Green tea decreased plasma levels of TG, and LDL cholesterol, reduced hepatic lipid accumulation, and body fat mass, and increased plasma HDL-C levels (Lukitasari et al., 2020; Saifur Rohman et al., 2021). 

Furthermore, green tea extract and EGCG reduced metabolic disorders caused by bisphenol A by acting as antioxidants and anti-inflammatory agents, regulating lipid metabolism, and improving insulin signaling (Mohsenzadeh et al., 2021).

It was reported that both green tea and EGCG-treated rats with experimental metabolic syndrome had lower TG levels. However, EGCG-treated animals had higher levels of HDL and PPARα gene expression than green tea-treated animals (Lukitasari et al., 2018). There is growing evidence that adiponectin and its receptor are key metabolic syndrome biomarkers (Ghadge et al., 2018). Animals with experimental metabolic syndrome treated with green tea extract had higher adiponectin levels (Nugroho et al., 2018). Daily intake of green tea extract (including 440 mg of EGCG and 180 mg of ECG) for 8 weeks, reduced weight gain and body mass index and lowered lipid peroxidation in obese patients with metabolic syndrome (Basu et al., 2010). The metabolic parameters and inflammatory biomarkers of these patients were the same as those of controls (Basu et al., 2011). 

Our results showed that olanzapine administration increased SBP, which decreased following ECG and EGCG treatment. Green tea administration for eight weeks also decreased SBP, blood sugar, TG, and total cholesterol, and increased HDL in women with metabolic syndrome (Mortazavi et al., 2018). However, in another clinical study on the Japanese population, green tea consumption did not affect blood pressure, plasma sugar, and lipid profile (Hino et al., 2007).

Leptin is a cytokine-like peptide generated by adipose tissue and controls food intake, body weight, and fat mass. Like our findings, olanzapine treatment increased leptin levels in rats (Minet-Ringuet et al., 2007; Zugno et al., 2012). On the other hand, both ECG and EGCG reduced leptin levels enhanced by olanzapine. In agreement with our findings, schizophrenia patients treated with olanzapine exhibited higher serum leptin levels, while adiponectin levels did not change (Hosojima et al., 2006; Tsuneyama et al., 2016). There is a positive correlation between plasma concentration of leptin and adiposity in healthy individuals and obese patients. It also directly affects insulin secretion. Furthermore, leptin levels have been associated with changes in blood glucose levels and weight gain during olanzapine treatment. Among the key pathophysiological aspects of metabolic syndrome is insulin resistance. As a result of olanzapine administration, FBS dramatically increased, which was then reduced by ECG and EGCG treatment. Various studies have shown that olanzapine is linked to high blood sugar levels and increased diabetes mellitus risk (Kumar and Thomas, 2011; Tanaka et al., 2008). Accordingly, chronic olanzapine administration led to hyperinsulinemia and elevated glucose levels in mice (Coccurello et al., 2006). Lukitasari and colleagues showed that green tea administration had an insignificant blood pressure‑lowering influence in the high sucrose/high-fat model of metabolic syndrome (Lukitasari et al., 2018; Lukitasari et al., 2020). In line with our findings, EGCG treatment attenuated insulin resistance and blood glucose levels in C57BL/6J mice consuming a high-fat/Western-style diet (Chen et al., 2011). EGCG treatment also prevented inflammation-induced β cell death by inhibiting mitochondrial membrane potential and suppressing reactive oxygen species generation, as well as preventing cytochrome c release. It is known that obesity, diabetes, insulin resistance, and hypertension cause endothelial dysfunction (Legeay et al., 2015). It was indicated that EGCG restored endothelial function and lowered blood pressure in spontaneously hypertensive rats. According to this study, EGCG stimulates endothelial nitric oxide production, possibly contributing to its beneficial effects on hypertension (Potenza et al., 2007). We also found that olanzapine reduced locomotor movement in the open field test which is in accordance with previous findings (Albaugh et al., 2011; Weston-Green et al., 2011). As Coccurello and coworkers did not observe any changes in motor activity in female mice exposed to olanzapine, the effect of olanzapine on motor activity could be related to sex (Coccurello et al., 2006). We showed that co-administration of ECG and EGCG with olanzapine reversed the hypolocomotion elicited by olanzapine. In agreement, administration of green tea extract with olanzapine significantly improved motor movements decreased by olanzapine (Razavi et al., 2017). Green tea also enhanced muscle mass and reduced fat stores in mice (Michna et al., 2003). It is worth mentioning that hypolocomotion contributes to obesity maintenance under long-term treatment with atypical antipsychotics (Zhang et al., 2014).

Based on the present study, olanzapine administration resulted in weight gain, dyslipidemia, hypertension, increased leptin levels, high blood sugar, and motor impairment. By administering both ECG and EGCG, olanzapine-induced metabolic changes were alleviated. EGC (20 mg) showed much more potential than EGCG (20 mg) in the reduction of olanzapine weight gain, TG, FBS, and leptin levels. This dose of EGC enhanced HDL higher than EGCG while locomotor activity increased more with EGCG. Other parameters did not differ between these catechins. According to these findings, green tea consumption may ameliorate some olanzapine side effects. The justification, however, requires well-controlled clinical studies. 
